# Residential Mobility, Family Structure, and Completion of Upper Secondary Education – A Registry-Based Cohort Study of the Norwegian Adolescent Population

**DOI:** 10.3389/fpsyg.2019.02311

**Published:** 2019-10-15

**Authors:** Tommy Haugan, Arnhild Myhr

**Affiliations:** ^1^Faculty of Nursing and Health Sciences, Nord University, Levanger, Norway; ^2^Trøndelag R&D Institute, Steinkjer, Norway; ^3^Department of Neuromedicine and Movement Science, Faculty of Medicine and Health Sciences, Norwegian University of Science and Technology, Trondheim, Norway

**Keywords:** upper secondary education, school completion, educational achievement, residential mobility, adolescence, parental education, family structures, social inequality

## Abstract

**Background:**

Longitudinal studies exploring the complex interplay between family structures and residential mobility on educational achievement and failure are lacking. We investigate the interplay between the number of residential moves during late childhood, parental education level, family living situation, and the probability of completing upper secondary education.

**Methodology:**

Detailed longitudinal data for a random sample of 30% of the entire Norwegian population born 1982 to 1989 (*N* = 121,247) and information on all their relocations between Norwegian enumeration districts from ages 10 to 18 years were extracted from the Norwegian population registries. Family structures were grouped into four intersectional family strata defined by combining categories of parental education level (distinguishing poorly educated and well-educated families) and the family’s living situation (comparing non-intact families with intact families). We applied two-level logistic regression models, which incorporated individual and family contextual factors, to estimate possible differences in completion rates of upper secondary education.

**Results:**

Non-completion of secondary education (which constitutes 29% of the study sample) increases incrementally with the number of residential changes across all four family structures, but this effect was not distributed evenly between the different family strata. Individuals in “well-educated, intact families” seem to be least affected by residential moves. On the other hand, the highest disadvantage of frequent moves was among adolescents in the stratum “poorly educated, intact families.” In poorly educated families the probabilities of completing secondary school among non-intact and intact families converge toward each other as the number of moves increase. About 43% of the variation in school completion may be attributed to differences between families. The highest risk of school non-completion was found among adolescents in poorly educated families, which accounted for 74% of the non-completers.

**Conclusion:**

We demonstrated underlying links between residential mobility and family structures on non-completion of upper secondary education. The adverse effect of frequent moves calls for attention in schools, public health agencies, and housing policies. The findings should be considered in a life course perspective, as the accumulation of unfavorable conditions during childhood and adolescence tends to constrict future prospects in terms of health and quality of life.

## Introduction

Educational inequality, which exists in most wealthy nations, is of global concern (OECD, 2018). Family origin and a family’s resources and strategies create a powerful social context that to a large extent predicts educational achievement (Corrás et al., 2017). Factors shown to be associated with youth non-completion of secondary education include low parental socioeconomic status (SES) and family adversity such as parental divorce, household instability, and poverty (Amato, 2001; De Ridder et al., 2013; Myhr et al., 2017). Adolescents who drop out of secondary education are substantially reducing their odds of having long, happy, and healthy lives (Viner et al., 2012). School dropout increases risk of long-term socioeconomic marginalization (Bäckman et al., 2015; OECD, 2018), unemployment (Caspi et al., 1998), dependence on public benefits early in life (De Ridder et al., 2012; Myhr et al., 2018), and mental and physical health problems (Marmot and Bell, 2012; Viner et al., 2012).

Internal migration – changing residence within national borders – is often voluntary but may be born of necessity. Northern and western Europe are characterized by high internal migration (Bell et al., 2015; Bernard, 2017). Various life course events impact residential mobility over the life span (Warner and Sharp, 2016). Young adults and families with children are the most frequent movers (Bell, 1996; Bernard, 2017; Morris et al., 2018), and children from single-parent households are even more likely than children from two-parent families to move (Feijten and Van Ham, 2007; Murphey et al., 2012). Parents may seek to relocate in pursuit of upward social mobility and access to better schools or neighborhoods (DeLuca and Dayton, 2009). Neighborhood and residential contexts affect individuals’ cognitive development, health, and educational achievement in heterogeneous ways and life course outcomes related to changes in such contexts may thus be highly individual (Sampson et al., 2002; Sharkey and Faber, 2014). Residential mobility in childhood interacts at the neighborhood, family, and individual levels in cumulative and compounding ways and thereby affect well-being and behavior through adolescence (Jelleyman and Spencer, 2008). Although one may assume both advantages and disadvantages from such transitions, moving itself is a potential source of stress for children, independent of any increase or decrease in residential quality or their origins and destinations (Jackson and Mare, 2009). Emerging research indicates residential mobility has a wide range of potential negative effects on children, including developmental, behavioral, and emotional problems (Oishi and Schimmack, 2010; Fowler et al., 2015; Webb et al., 2016), a higher risk of substance abuse and violent offending (Haynie and South, 2005; Fowler et al., 2015; Webb et al., 2016), and poor academic achievement (Voight et al., 2012).

However, it is not clear that residential mobility has only negative effects on child development. Methodological issues that produce inaccurate estimates might lead researchers to overestimate the deleterious effects of moving by confounding it with various factors (Garboden et al., 2017). Research that distinguishes the circumstances shaping residential mobility shows that its detrimental effects on children are more likely to emerge when it occurs in difficult circumstances [see, e.g., (Fantuzzo et al., 2004; Hanushek et al., 2004)], and could also be beneficial to health over the longer term (Morris et al., 2018). A 10-year follow-up study in the United States found a negative association between the number of childhood residential moves and well-being as adults among introverts but not among extraverts (Oishi and Schimmack, 2010). The authors suggest that residential moves can be a risk factor for introverts and that extraversion can be an interpersonal resource for social relationships and well-being in mobile societies. Some studies isolate the issue of family structure, finding that the presence of both (biological) parents prevents harm through frequent moving, while frequent movement among children of single or remarried parents may result in adverse school performance (Tucker et al., 1998; Scanlon and Devine, 2001). The combination of both school and family transitions might increase children’s risk of social withdrawal and isolation (Dupere et al., 2015). Likewise, adolescents who change both addresses and schools are often more likely to drop out of school, an effect that may function through disruptions in peer networks (South et al., 2007). Thus, the possible influence of household change and family SES should be accounted for when isolating the effects of residential instability for children and youth. The structural amplification theory states that unfavorable “social conditions decrease the likelihood of attaining personal resources that otherwise would moderate undesirable consequences” (Ross and Mirowsky, 2011). From such a perspective, residential mobility during adolescence might make it harder while residential stability might make it easier to attain personal resources (such as social networks) that counteract unfavorable family conditions.

Important questions regarding the complex interplay between family resources and residential mobility on school achievement and failure still remain unanswered. More social epidemiological studies where the diversity of human population movement are not reduced to a simple dichotomy (moved or not moved) during childhood and adolescence are needed. Longitudinal studies accounting for the number of residential moves while growing up may provide a more comprehensive picture of how family resources and strategies shape educational inequalities. In the present study, we explore the interplay between number of residential moves during late childhood, parental education level, family living situation, and the probability of completing upper secondary education. We hypothesize that level of parental education and family living situation condition the association between residential mobility and school completion.

## Materials and Methods

### Data Sources

This study is based on national administrative data from Statistics Norway’s event database, FD-trygd (Akselsen et al., 2007) and the Norwegian National Education Database (NUDB; StatisticsNorway, 2001) during the period 1992 to 2010. The FD-Trygd database assembles event registration data for all Norwegian citizens from several official administrative and statistical registers and includes life cycle events and demography, work status, income, and national insurance status. We extracted a random sample of 30% (*N* = 161,743) of all Norwegians aged 21 to 27 years in 2010 (i.e., born in the period 1982 to 1989), stratified by age, gender, and municipality of residence. This cohort gave us long enough follow-up periods to predict the effect of residential mobility during childhood and adolescence (from age 10 until age 18) on completion of upper secondary education. This dataset is linked to the NUDB database by using the unique 11-digit personal identification numbers assigned to all Norwegian citizens. Through a unique family identification code attached to each personal identification number, we were also able to allocate information on the parents and the household to each individual. This enabled us to map the parental education level and to determine whether the individual lived with his or her parents. Hence, we ended up with linked longitudinal data for both subjects and their parents, including annual updates on residential identifiers, parental education level, social and financial insurance status, and the family’s living situation. From the 161,743 individuals initially included in the dataset, we excluded 13,745 individuals (7.5%) from the sample due to missing their educational data at age 21. In addition, 2,523 individuals were excluded due to unknown parental identity and 24,228 individuals due to missing residential identifiers during the follow-up period. A large majority, 97%, of these excluded individuals immigrated to Norway during the study’s follow-up period (i.e., 1992–2010). Thus, to ensure equal observation time (i.e., from age 10 until age 18) for all subjects in the study sample, these individuals were excluded from the study. The final dataset contained 121,247 individuals.

### Assessment of Variables

Several social dimensions influence educational achievement and may contribute to generate educational inequalities (Støren and Helland, 2009; Bäckman and Nilsson, 2010; Myhr et al., 2017). We have selected the following demographic and socioeconomic determinants to evaluate their effects on the probability of completing secondary education among the Norwegian youth population.

### Individual Level

#### Non-completion of Secondary Education

The binary dependent variable is whether (or not) the individual completed upper secondary education by age 21, obtained from the NUDB database. In Norway, where education is by and large public, young people generally begin upper secondary education at age 16, and it consists primarily of a high school academic track of 3 years and/or vocational education, which lasts between 2 and 4 years (Ministry of Education and Research, 2007). We examined completion rates 5 years later, i.e., at age 21. The completion rate for upper secondary education in Norway has remained stable at around 70% since the country’s major education reform in 1994, with slightly higher completion rates in recent years (StatisticsNorway, 2018).

#### Gender

We categorized gender as male or female and used male as the reference category.

#### Residential Mobility

Residential mobility was measured as the number of moves between Norwegian neighborhoods while the children were between the ages of 10 and 18 years. For each follow-up year only one move was counted, which means that the maximum possible number of residential moves during the observational period was nine. We used the individual’s recorded census enumeration district, which is the lowest geographical level for Norwegian population statistics, to identify their neighborhoods (Akselsen et al., 2007).

### Family Level

The unit of analysis at the second level is the families (*N* = 110,865) identified in the study sample. Unique family identifiers enable us to identify siblings in our 30% random study sample who shared the same mother and father as well as to link information on children to that of their mother and father.

#### Parental Education Level

Parental education level, obtained from the NUDB database, was based on the Norwegian standard classification of education (StatisticsNorway, 2001), providing nine levels which were collapsed into two education level groups: (i) both parents completed upper secondary or tertiary education, termed as “well-educated”; and (ii) neither or only one parent completed upper secondary education, termed as “poorly educated.”

#### Family Living Situation

The individual’s living situation was grouped into one of two categories, defined as (i) “intact family”: living with two registered parents at both age 10 and age 16; or (ii) “non-intact family”: living with only one (or no) parent at age 16.

#### Family Structure

In order to consider simultaneously multiple axes of inequality we created four intersectional strata, corresponding to combinations of parental education level (two categories) and family living situation (two categories). The intersectional strata, referred to as “family structure,” were divided into the following categories: (i) “well- educated intact family,” (ii) “well-educated non-intact family,” (iii) “poorly educated intact family,” and (iv) “poorly educated non-intact family.”

#### Family Poverty

The dichotomous variable “family poverty” was defined as having parents receiving social security benefits in the period from 10 to 16 years of age (according to the indexed person’s age).

### Statistical Methods

We investigate the relationship between completion of secondary education and number of residential moves during late childhood and test the hypothetical interaction with family structure by using two-level logistic regression analysis. The data have a two-level hierarchical structure with individuals (Level 1, *n* = 121,247) nested within families (Level 2, *n* = 110,865). The family context may condition individual level variation in completion of upper secondary education due to unmeasured factors. We therefore fitted a two-level random intercept model (Goldstein, 1995; Rabe-Hesketh and Skrondal, 2012; Snijders and Bosker, 2012) to distinguish the individual and family sources of variation in the outcome.

We modeled the prediction of school completion in five steps. First, we estimated an “empty” model, which includes only a random intercept, representing the variation in school completion between the two initial levels. This allowed us to determine the impact of the family context on the outcome (Merlo et al., 2005). Model 2 (in Table 2) includes gender and residential mobility variables. In Model 3, we adjust for the family predictors (i.e., family structure and poverty). Model 4 adds the interaction terms residential mobility and family structure. To estimate the family level variance we need to have multiple children per family (Rasbash et al., 2010). Since most individuals in the present study were in family groups of only one child, the variance at the family level for these individuals included the individual variance. To account for this in the analysis, we also estimated the family variance only for those families in the study sample with more than one child (Rasbash et al., 2010; Dundas et al., 2014). Finally, in Table 3, the random intercept logit model was extended for the relationship between residential mobility and school completion to allow residential mobility effect to vary across families. We fitted a two-level random slope model (i.e., individuals nested within families) in order to examine whether the relationship between residential mobility and school completion varies between families. We used a likelihood ratio test (LR test) to compare the random intercept and the random slope model’s goodness of fit (Rabe-Hesketh and Skrondal, 2012).

Estimates for fixed effects are reported as odds ratios (OR) with 95% confidence intervals (CI). The relative importance of the general family contextual effects is assessed by the variance (on the log odds scale) with 95% CI and the intra-class correlation coefficients (ICCs; Snijders and Bosker, 2012). The ICC measures the correlation in the outcome of “school completion” between two individuals randomly selected from the same family. The larger the ICC, the stronger the clustering in school completion within the family and the larger the general family contextual effects. The multilevel regression model parameters were estimated by using the mixed effects method using STATA/MP software (version 13).

### Ethics Statement

Statistics Norway constructed the study sample with linked longitudinal data for both the subjects and their parents, by means of record linkage of different registries integrated into the Statistics Norway database by using the unique Norwegian personal identification number. Finally, Statistics Norway delivered the data to us without personal identification numbers to ensure the anonymity of the study subjects. The study and the data linkage procedures were approved by the Regional Committee for Medical and Health Research Ethics of Mid-Norway (permission 2011/783).

## Results

### Descriptive Statistics

Table 1 presents descriptive information for the children and their parents among completers and non-completers of upper secondary education. Non-completers comprised 29% of the sample, which is in accordance with Norwegian official statistics (Chaudhary, 2011). In this study population, the highest absolute number of individuals belong to the family structure stratum “poorly educated intact family,” where we also identified the highest absolute number of non-completers.

**TABLE 1:** Characteristics of children and their parents by whether or not the children completed upper secondary education by age 21.

**Variable**	**Non-completers**		**Completers**	
	**(*N* = 35,254, 29.1%)**		**(*N* = 85,993, 70.9%)**	
	***N***	**%**	***N***	**%**
Individual level variables				
Female	14,504^a^	41.1	44,630	51.9
Years with residential mobility	0.917^b^	1.279	0.475	0.878
10–18 years (mean, SD)				
Residential mobility (categorical)				
Never moved at age 10–18 years	18,767^a^	53.2	60,012	65.0
1 year with move	7,833^a^	22.2	16,257	19.9
2–3 years with move	6,744^a^	19.1	8,491	12.6
≥4 years with move	1,910^a^	5.4	1,233	1.4
Family level variables				
Family structure				
Well-educated intact family	5,287^a^	15.00	31,194	36.28
Well-educated non-intact family	3,740^a^	10.61	8,623	10.03
Poorly educated intact family	14,027^a^	39.79	34,206	39.78
Poorly educated non-intact family	12,200^a^	34.61	11,970	13.92
Family poverty	10,424^a^	29.57	8,490	9.87
Father’s identity unknown	676^a^	1.92	694	0.81

*^*a*^Significant difference (*p*-value ≤ 0.05) between groups tested by chi square test. ^*b*^Significant mean difference (*p*-value ≤ 0.05) between groups tested by independent sample *t*-test.*

In total, 53% of non-completers and 70% of the school completers had never moved their official residence when they were 10 to 18 years old. In total, 32% of the adolescents in the study population moved to another neighborhood in one to three of the nine observational years, and about 3% moved in four or more. The mean number of years with move in the observational period for the non-completers was almost one, which is almost twice the mean number for completers (see Table 1).

### The Impact of Childhood Residential Mobility, Family Structure, and Their Interactions on the Completion of Secondary Education

The prevalence of school dropout at the family level differs. Keeping only the second random intercept in the model (Model 1 in Table 2), we found that the ICC is 0.43. In other words, the empty model suggests that about 43% of the variation in school completion could be attributed to differences between families. Models 2 and 3 in Table 2 display the observational associations with school completion and residential mobility (Model 2), adjusted for family structure, and poverty during childhood (Model 3). The highest risk of school non-completion was found within poorly educated families. Having a non-intact family was also shown to be a potent risk factor. However, adolescents living in poorly educated but intact families have overall lower odds for school completion than their counterparts living in well-educated but non-intact families. Anyway, adolescents with poorly educated non-intact families struggled the most with school completion. The odds of school completion is about 80% lower for this group compared to well-educated intact families. Moreover, females had almost twice as high a likelihood to complete upper secondary school compared to males, while family poverty was estimated to increase the risk of school dropout by 63%.

**TABLE 2:** The impact of residential mobility and its interaction with family structure (education level and family living situation) on the probability of completing upper secondary education.

	**Model 1**		**Model 2**		**Model 3**		**Model 4**	
	**OR**	**95% CI**	**OR**	**95% CI**	**OR**	**95% CI**	**OR**	**95% CI**
**Fixed effects**								
Female			1.884	1.81–1.96	1.857	1.79–1.93	1.857	1.79–1.93
Frequency of household mobility			0.584	0.57–0.60	0.778	0.76–0.79	0.794	0.76–0.83
Family structure								
Poorly educated non-intact family					Ref		Ref	
Poorly educated intact family					1.742	1.66–1.83	1.838	1.73–1.95
Well-educated non-intact family					2.431	2.28–2.60	2.496	2.29–2.73
Well-educated intact family					4.965	4.64–5.32	5.091	4.72–5.50
Family poverty					0.371	0.35–0.39	0.372	0.35–0.39
Father’s identity unknown					0.654	0.56–0.76	0.653	0.56–0.76
**Interaction family structure and residential mobility**								
Poorly educated non-intact family							Ref	
Poorly educated intact family							0.919	0.88–0.96
Well-educated non-intact family							0.981	0.94–1.03
Well-educated intact family							0.996	0.94–1.05
**Random effects**								
**Family variance (95% CI)**	2.571	2.2–2.88	2.207	1.95–2.50	1.520	1.31–1.76	1.519	1.31–1.76
ICC (%)	43.87		40.16		31.60		31.59	
−2log likelihood	145621.8		140199.6		132123.0		132106.0	
**Family variance > 1 child**								
**Family variance (95% CI)**	2.521	2.24–2.84	2.187	1.93–2.48	1.495	1.28–1.74	1.496	1.29–1.74
ICC (%)	43.39		39.93		31.25		31.25	
−2loglikelihood	46817.0		45441.3		42725.1		42701.0	

In Table 3, we extended the random intercept logit model to examine whether there are differences between families in the relationship between residential mobility and probability of completing upper secondary school. The two-level random intercept model, which is nested in the random slope model, is rejected at the 5% significance level (using a likelihood ratio test), suggesting that the impact of residential mobility on school completion does vary between families.

**TABLE 3:** Parameter estimates and log-likelihood values for the random intercept and random slope logistic regression models.

	**Random intercept**		**Random slope**	
			**(coefficient)**	
**Parameter**	**Coef**	**SE**	**Coef**	**SE**
**Individual level**				
Intercept	1.5761	1.53–1.62	1.5729	1.52–1.62
Residential mobility	–0.5233	−0.55 to −0.50	–0.5334	−0.56 to −0.51
**Family level random part**				
Residual variance	2.1392	1.89–2.42	2.0617	1.81–2.35
intercept				
Residual variance slope			0.1032	0.06–0.19
−2Log likelihood	141505.4		141488.7	
BIC	141540.5		141535.5	
AIC	141511.4		141496.6	

*Likelihood ratio test: LR chi2 = 16.77, *p*-value < 0.0001.*

**FIGURE 1:**
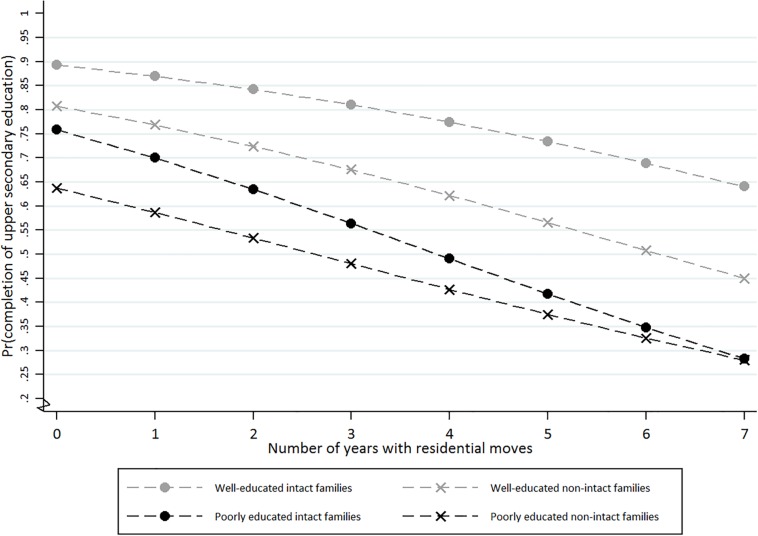
Predicted probability (Pr) of upper secondary school completion by different types of family structures – divided into (i) “well-educated and intact,” (ii) “well-educated but non-intact,” (iii) “poorly educated but intact,” and (iv) “poorly educated and non-intact” – and number of years with residential moves during the follow-up period from 10 to 18 years old.

Children whose families did not move and who lived in a well-educated intact family had a 89% chance of completing upper secondary school, compared to 81, 76, and 64% for residential stayers who were living in a well-educated non-intact family, a poorly educated intact family, and a poorly educated non-intact family, respectively. Figure 1 shows that the predicted probability of school completion decreases incrementally with the number of years with residential moves. In general, for every additional year with a residential move the probability of school completion decreased by 26%. However, well-educated intact families seem to be least affected by residential moves, and among children in poorly educated intact families the adverse effect of moves is significantly steeper than for the other three family structures (Figure 1). For movers who changed residential household in three out of the nine follow-up years, for example, the predictive probability of school completion was 81% in a well-educated intact family, 68% in a well-educated non-intact family, 56% in a poorly educated intact family, and 48% in a poorly educated non-intact family.

In poorly educated families the probabilities of school completion within non-intact and intact families converge toward each other as the number of moves increase. This result was not evident among well-educated families, and in fact they seem to grew more distinct.

For frequent movers the negative impact of a non-intact family situation on school completion appeared to be most evident among well-educated families, whereas among non-movers the adverse impact of non-intact families was most prominent among poorly educated families (see Figure 1).

## Discussion

This registry-based cohort study reinforces the relevance of the family context and the complex interplay between family structures and residential mobility on the probability of completing upper secondary education. Our parametric estimations indicate that the risk of school non-completion increases for each additional year with residential move during the period from 10 to 18 years of age. The negative impact of frequent residential mobility in school completion differs, however, depending on the family’s structure, as measured by parental education level and family living situation. Our two-level model estimated that about 43% of the variation in school completion can be attributed to the differences between families. Considering the significant impact of family belonging, it is essential to uncover the risk factors at the family level and their moderators. Among those who did not complete upper secondary education (about 29% of the study sample), 74% have poorly educated parents, which is in line with other Norwegian intergenerational studies (De Ridder et al., 2013; Myhr et al., 2017).

The impact of coming from a non-intact family affected children’s likelihood of completing school – although not as much as the parental education level. In our study sample, three out of 10 adolescents were living with only one parent at age 16, which accounted for about 45% of the school non-completers. Previous studies have shown that adolescents not living with both parents are less well-adjusted psychologically and socially; they are exposed to a lower family income and they have lower academic achievement, relative to adolescents from intact families (Amato, 2001; Seijo et al., 2016). The explanation as to why negative outcomes are most common among children in single-parent families might be due to both a selection process (i.e., pre-existing differences) and a causal relationship (i.e., negative effects are the consequence of parental separation) (Seijo et al., 2016). Whatever the underlying cause, these negative outcomes are concerning because the proportion of children and adolescents living in single-parent families, particularly in Western countries, is growing (Child Trends, 2015).

At the same time, our study suggests that residential stability can at least partially compensate for the negative impact of family disruption and low parental education level. Our findings conform to others that show links between number of total moves and adverse outcomes in health and well-being through maturity and later in life (Webb et al., 2016; Morris et al., 2018). That is, they show elevated risks across the socioeconomic spectrum (Webb et al., 2016). In our study, 47% of the participants had one or more years with residential moves between the ages of 10 and 18 years. The risk of non-completion of secondary school increased incrementally with the number of residential changes across all family structures. In other words, each additional residential move between ages 10 to 18 years lowered the likelihood of upper secondary school completion. But this effect was not distributed evenly between the different family strata, in a dose-response fashion. The well-educated intact families seem to be least affected by residential moves, where even frequent movers had a high predictive probability of school completion. This observed trend is in line with the idea behind the resource substitution and structural amplification theory in the sense that residential stability is to a certain extent most valuable among adolescents living in family structures that can be unfavorable. Residential mobility may cause unstable social conditions during childhood and adolescence and decrease the likelihood of attaining personal resources, such as social networks and long lasting friendships, that otherwise would moderate undesirable family conditions related to poorly educated parents and/or family disruption. Children are vulnerable to damaged networks and environments as a result of residential relocations (Morris et al., 2018). Residential mobility may threaten child development through mechanisms such as changes in school and peer networks (Coulton et al., 2007). Household moves may also disrupt connections with parents and extended family. Well-educated, intact families have higher social and socioeconomic resources that might prevent loss of the children’s social capital upon residential relocation, thereby minimizing the unfavorable consequences of household moves (Hagan et al., 1996).

However, the underlying links between residential mobility and family structures on non-completion of upper secondary education are intricate and can be difficult to fit into overall education and health mobility (sociological and psychological) theories and models. We found that adolescents living in poorly educated, intact families showed the most significant disadvantage of household moves. Among children of poorly educated families, the likelihood of upper secondary school completion within intact and non-intact families converge toward each other as the residential moves increase. This finding is puzzling given previous studies suggesting that frequent residential moves are a marker for family dysfunctional and chaotic households (Boynton-Jarrett et al., 2013). We would therefore expect that the non-intact families experience the greatest challenges with residential mobility, regardless of education level. However, it might be that residential moves within intact, poorly educated families are more often related to adverse circumstances such as economic difficulties and work situation than the nature of mobility among other family structures. Additionally, residential mobility may indicate that adolescents living in poorly educated, non-intact families have less social capital to lose by residential relocations and more often, and to a greater extent, a relocation actually represents an opportunity to restart one’s social network and environmental adaptation. A life course approach should be adopted given the time lag between household mobility during childhood and outcomes related to education and health. To offer more conceptual understanding of residential mobility, Morris et al. (2018, p. 123) stated that *“a greater focus on mobility as a biography that is taken into account alongside other life events will permit a “bigger picture” view of mobility.”*

Overall, the present study conforms with past research showing that residential mobility is a crucial factor in determining educational inequalities (Haelermans and De Witte, 2015). Frequent moves may accumulate unfavorable personal and social conditions that make it more difficult to attain personal resources necessary to complete upper secondary education (South et al., 2007; Metzger et al., 2015). Associations between residential mobility and problem behaviors could, however, be driven by school mobility and not necessarily the move itself (Gasper et al., 2012). Nevertheless, children have little influence over mobility decisions, which may imperil their existing social networks. Residential moves require, in many cases, children to change schools. Our study’s definition of moves makes changing school nearly certain, although upper secondary school affiliation may be the least likely to change as a result of such moves. The impact of switching schools on dropout varies depending on a youth’s initial risk for switching schools (Gasper et al., 2012). To the extent that our study addresses moves that lead to changing schools, it must be taken into consideration when the findings are to be interpreted.

The notation that non-completion of secondary education increases incrementally with the number of residential changes during childhood could be considered in light of basic psychological needs. Relationships and a sense of connectedness play a critical role in promoting well-being in the context of schools (Graham et al., 2016). The desire for interpersonal attachments – the need to belong – is considered a fundamental human motivation and a basic psychological need (Baumeister and Leary, 1995). Baumeister and Leary (1995) propose that human beings need a few close relationships, and we need these interactions to occur in a framework of long-term, stable caring and concern, and when the need for belonging is satisfied, positive social, behavioral, and psychological outcomes can be achieved. The authors also state that forming additional bonds beyond those few persons has less and less impact on emotional and cognitive outcomes. However, Bronfenbrenner’s ecological theory of human development and socialization – also called the bioecological systems theory – suggests a broader framework by underlining the influence of different levels and sizes of social and cultural environments on human development (Bronfenbrenner, 1979; Bronfenbrenner and Morris, 2006). When a child experiences a residential move, many of the child’s closest surroundings and nearest relationships, like teachers and classmates at school, hobby club mates, the neighbors and the connections between the settings are interrupted, and new relationships need to be built up. Consequently, these children are exposed to a socially vulnerable situation. Thus, initiatives that promote social inclusion in the school, but also in other “microsystem” arenas, such as family, neighborhood, peers, and sport clubs, are important to prevent loss of social capital when adolescents change residence.

In a recent methodological review of the residential mobility literature, the concept of mobility is inconsistently operationalized along four dimensions: school vs. residential, distance, timing, and frequency (Garboden et al., 2017). The authors therefore call for an ideal mobility module that collects “full residential and school trajectories of children, including any instigating events and contemporaneous changes in family structure” (Garboden et al., 2017, p. 258). Given that we were not able to do this, the current study has several limitations and the findings are vulnerable to selection bias. A major limitation is the lack of information about the reasons for residential mobility decisions. We did not take into account ethnic background or separate the educational levels of mother and father. A previous study from Norway found that ethnic majority students benefit the most from having parents with high education, and further that minority girls largely benefit from their mother’s education level (Støren and Helland, 2009). Further studies with specific analyses that reveal interaction effects are needed to give a more nuanced explanation of the complexity between individual characteristics, family background, resources and living arrangements, and school completion. Regarding measure of family SES, there are several other indicators than parental education level that can be used such as family income, professional status, parental financial wealth, and receipt of social security benefits. Moreover, an ideal mobility study includes explanatory variables at multiple appropriate levels and allows the levels (e.g., the context) to change over time. A highly relevant level in the present study is the school or schools the children attended during the follow-up period. Thus, it would be beneficial to analyze the data by multiple membership cross-classified multilevel models that allow the neighborhood, school, and family levels to change over time (Chandola et al., 2005; Chung and Beretvas, 2012; Leckie, 2013).

Resource substation theory of education and health outcomes later in life suggests that persons with disadvantaged family backgrounds benefit the most from educational attainment (Ross and Mirowsky, 2011; Schaan, 2014). Thus, the variation in school completion between family structures (in our study defined by parental educational level and residing with one or both parents) plays an important role in public health efforts. Further research in this area should emphasize the underlying interplay between residential mobility and family resources on non-completion of upper secondary education. In our study, a high proportion of non-completers live with poorly educated parents. This is in accordance with other studies showing that parental educational attainment, to a large extent, structures the education level of their offspring. Thus, future public health efforts should promote intergenerational educational mobility. The adverse effect of frequent moves, particularly among adolescents in poorly educated families, calls for attention in schools, family, public health agencies, and housing policies to promote stability and sustainable life situations among vulnerable families. Adolescents in poorly educated and non-intact families are particularly at risk for school non-completion and should therefore be given priority in future efforts to increase completion rates. Various stakeholders have to communicate and collaborate in their recognition of the importance of psychological membership and the concept of belonging. The stakeholders should build productive relationships on multiple levels of practice to support children with social, emotional, and behavioral difficulties in school contexts (Botha and Kourkoutas, 2016). Our findings should be considered in a life course perspective, because accumulation of unfavorable conditions during childhood and adolescence tend to constrict future prospects in terms of health and quality of life.

## Data Availability Statement

Due to the legislation governing scientific ethics, the data that support the findings of this study are only available on request in accordance with the agreement with the owner of the data, Statistic Norway, and the approver of the study, the Regional Committees for Medical and Health Research Ethics (REC) in Mid-Norway. Please see http://www.ssb.no/en/omssb/tjenester-og-verktoy/data-til-forskning for the procedure and requirements to obtain microdata from Statistic Norway.

## Ethics Statement

The present study is based on retrospective analysis of registry data. The Regional Committees for Medical and Health Research Ethics (REK) of Mid-Norway approved the study and the data linkage procedures (permission 2011/783). The ethic committee REK formally waived the need for consent. The exemptions were given because our study used data registries where the information was collected from sources other than the persons themselves.

## Author Contributions

TH and AM designed and planned the study, and interpreted the results. AM structured and analyzed the data, and assisted with writing and editing of the manuscript. TH had primary responsibility for the writing and editing of the manuscript. Both authors took responsibility for the integrity and accuracy of the data analysis and the decision to submit this manuscript for publication, and read and approved the final manuscript.

## Conflict of Interest

The authors declare that the research was conducted in the absence of any commercial or financial relationships that could be construed as a potential conflict of interest.
